# Antibacterial activity of aquatic gliding bacteria

**DOI:** 10.1186/s40064-016-1747-y

**Published:** 2016-02-04

**Authors:** Yutthapong Sangnoi, Theerasak Anantapong, Akkharawit Kanjana-Opas

**Affiliations:** Department of Aquatic Science, Faculty of Natural Resources, Prince of Songkla University, Hat Yai, Songkhla 90110 Thailand; Department of Industrial Biotechnology, Faculty of Agro-Industry, Prince of Songkla University, Hat Yai, Songkhla 90110 Thailand

**Keywords:** Aquatic gliding bacteria, Antibacterial activity, Pathogenic bacteria, Isolation media, Agar-well diffusion assay, Biofilm, Novel genus

## Abstract

The study aimed to screen and isolate strains of freshwater aquatic gliding bacteria, and to investigate their antibacterial activity against seven common pathogenic bacteria. Submerged specimens were collected and isolated for aquatic gliding bacteria using four different isolation media (DW, MA, SAP2, and Vy/2). Gliding bacteria identification was performed by 16S rRNA gene sequencing and phylogenetic analysis. Crude extracts were obtained by methanol extraction. Antibacterial activity against seven pathogenic bacteria was examined by agar-well diffusion assay. Five strains of aquatic gliding bacteria including RPD001, RPD008, RPD018, RPD027 and RPD049 were isolated. Each submerged biofilm and plastic specimen provided two isolates of gliding bacteria, whereas plant debris gave only one isolate. Two strains of gliding bacteria were obtained from each DW and Vy/2 isolation medium, while one strain was obtained from the SAP2 medium. Gliding bacteria strains RPD001, RPD008 and RPD018 were identified as *Flavobacterium anhuiense* with 96, 82 and 96 % similarity, respectively. Strains RPD049 and RPD027 were identified as *F*. *johnsoniae* and *Lysobacter brunescens*, respectively, with similarity equal to 96 %. Only crude extract obtained from RPD001 inhibited growth of *Listeria monocytogenes* (MIC 150 µg/ml), *Staphylococcus aureus* (MIC 75 µg/ml) and *Vibrio cholerae* (MIC 300 µg/ml), but showed weak inhibitory effect on *Salmonella typhimurium* (MIC > 300 µg/ml). Gliding bacterium strain RPD008 should be considered to a novel genus separate from *Flavobacterium* due to its low similarity value. Crude extract produced by RPD001 showed potential for development as a broad antibiotic agent.

## Background

Gliding bacteria are characterized as Gram negative, unicellular, swarm-colony-producing and uniquely motile by the ability to glide on surfaces. They are commonly found in various terrestrial, fresh, and seawater environments (Dawid 2000). These microorganisms play an important role in the degradation of plants and animal debris in many environments. The genera *Flavobacterium* and *Lysobacter* are gliding bacteria belonging to phyla *Bacteroidetes* and *Proteobacteria*, respectively. These genera are typically founded in plant, soil, and aquatic habitats. In recent years, isolation reports for *Flavobacterium* including *F*. *anhuiense*, *F*. *tiangeerense*, *F*. *ginsengiterrae*, *F*. *compostarboris*, *F*. *hauense*, *F*. *aquaticum*, *F*. *limnosediminis*, *F*. *longum* and *F*. *urocaniciphilum*, *F*. *panaciterrae*, *F*. *lacus*, and *F*. *faecale* have been released (Dong et al. 2013; Fujii et al. 2014; Jin et al. 2014; Kim et al. 2011, 2012, 2014; Lee et al. 2013; Li et al. 2014; Liu et al. 2008; Subhash et al. 2013; Xin et al. 2009). In addition, papers describing the isolation of new *Lysobacter* strains including *L*. *capsici*, *L*. *ximonensis*, *L*. *oryzae*, *L*. *soli*, *L*. *korlensis* and *L*. *bugurensis*, *L*. *arseniciresistens*, *L*. *oligotrophicus*, and *L*. *panacisoli* have been published (Aslam et al. 2009; Choi et al. 2014; Fukuda et al. 2013; Luo et al. 2012; Park et al. 2008; Srinivasan et al. 2010; Wang et al. 2009; Zhang et al. 2011). Despite the need for intensive isolation procedures, the screening of these two genera for antimicrobial properties was conducted for the purpose of potential antibiological applications. Genus *Flavobacterium* inhabits both terrestrial and aquatic environments. Several strains, such as *F*. *psychrophilum*, *F*. *columnare* and *F*. *spartansii* are known as fish pathogens (Cerro et al. 2010; Loch and Faisal 2014; Verma and Rathore 2013). However, few research studies investigating their biological activities have been published. The isolation of the β-lactam antibiotic deacetoxycephalosporin C, from *Flavobacterium* sp. SC 12,154 was reported by Singh et al. (1982). Another substance, monoacyldiglycosyl-monoacylglycerol was obtained from *F*. *marinotypicum* (Yagi and Maruyama 1999). The antimicrobial metabolites extracted from genus *Lysobacter* have been reported several times. Lysobactin, a potent antibiotic compound, was first isolated from *Lysobacter* sp. in 1988 by Bonner et al. (1988) and O’Sullivan et al. (1988). Tripopeptins were obtained from another strain of this microorganism in 2001 (Hashizume et al. 2001). Subsequently, strong antimicrobial metabolites isolated from the newly-discovered species *L*. *capsici* demonstrated abilities to inhibit the growth of bacteria, yeast, and fungi (Park et al. 2008). Moreover, *L*. *enzymogenes* C3 and *Lysobacter* sp. SB-K88 exhibited antagonistic activity against multiple plant pathogens (Islam 2010; Li et al. 2008). This indicated that *Lysobacter* spp. were bio-control bacteria capable of inhibiting plant diseases. Thus, the present study aimed to isolate and examine the antibacterial activity of aquatic gliding bacteria.

## Methods

### Isolation and purification

The submerged or moistened specimens including plant debris, algae, sediments, plastics, metal pieces, biofilms, and animal dungs were collected from the plant genetic resources conservation area at Cheow Lan Reservoir in Surat Thani province, Southern Thailand (GPS location: 8.5814 N 98.4830 E). Fifty-four specimens were subjected to a gliding bacterial isolation program using four different isolation media: DW (agar 15 g; distilled water 1 l), modified MA (peptone 5 g, yeast extracts 1 g, MgCl 9 g, NaSO_4_ 4 g, CaCl 2 g, agar 15 g, distilled water 1 l), SAP2 (tryptone 1 g, yeast extracts 1 g, agar 15 g, distilled water 1 l), and Vy/2 (baker’s yeast paste 5 g, agar 15 g, distilled water 1 l) (Sangnoi et al. 2009). Specimens were carefully cut into small pieces, then placed directly onto each type of isolation medium and incubated at room temperature. After the microorganisms displayed motility by gliding, the spread of the swarm colony was observed. After that, a clean edge of each swarm colony was sliced off and sub-cultured using another fresh isolation medium. Pure cultures were obtained by repeat sub-culturing 5–6 times. The pure strains were maintained on SAP2 agar medium at 4 °C for working strains, and fresh cells were preserved in 20 % glycerol at −20 °C for long term preservation.

### Morphological, physiological, and biochemical analysis

Cell morphology and gliding motility were observed using an Olympus BX50 light microscope with a drop of lactophenol blue added to enhance observational ability. A slide with thin SAP2 agar medium overlay was used to examine gliding motility. After placing a swarm colony on an overlay and allowing incubation, we observed gliding motility. Catalase activity was tested by bubble formation in 3 % H_2_O_2_ solution. Oxidase activity was determined by the oxidation of 1 % tetramethyl-p-phenylenediamine on filter paper. Acid production from carbohydrates was investigated using the commercial API 50 CH and API 20 E systems (BIOMERIEUX). Enzyme production was tested using the commercial API ZYM system (BIOMERIEUX), in accordance with manufacturer instructions.

## 16S rRNA gene sequence and phylogenetic analysis

The genomic DNA of the isolates was extracted using a Genomic DNA mini-kit (Geneaid) to determine phylogenetic relationships. The 16S rRNA genes were amplified by PCR using a set of 16S rRNA gene universal primers 27F (5′-AGAGTTTGATCATGGCTCAG-3′) and 1492R (5′-GGTACCTTGTTACGACTT-3′). The amplified PCR products were purified by GF-1 AmbiClean Kit (PCR/Gel) (Vivantis). Sequencing reactions were performed with the same universal primers, manufactured by First BASE Laboratories Sdn Bhd’s. Partial DNA sequences were compared with related sequences using the BLAST program, part of the GenBank/EMBL/DDBJ database (Altschul et al. 1990). Multiple alignments of the 16S rRNA gene sequences were carried out using the CLUSTAL_X program, version 1.83 (Thomson et al. 1997). Nucleotide substitution rates (*K*_nuc_ values) were determined (Kimura 1983), and the phylogenetic tree was constructed using the MEGA 5.22 program (Tamura et al. 2011). A bootstrap value test was performed with 1000 replicates, and a phylogenetic tree was determined using neighbor-joining, maximum-parsimony, and maximum-likelihood methods (Felsenstein 1985; Saitou and Nei 1987).

### Crude extract preparation

Each gliding bacterial isolate was cultivated in a 250 ml Erlenmeyer flask containing 100 ml of SAP2 broth medium and 2 g of amberlite XAD-16 resins (Sangnoi et al. 2008, 2009, 2014; Spyere et al. 2003). The cultivation flasks were incubated and shaken at 200 rpm for 7 days at 25 °C. The resins were collected using a nylon mesh and rinsed with deionized water to remove unwanted cells and other contaminants. Resins were left to dry at room temperature for 15 min and also soaked in 100 ml methanol for 1 h. Methanolic phases were separated from the resins, and methanolic crude extracts were obtained after evaporating a methanol solvent (Sangnoi et al. 2008, 2009, 2014). Crude extracts were prepared to a final concentration of 25 mg/ml in DMSO.

### Antibacterial assay

The agar-well diffusion assay as described by Wonghirundecha et al. (2014) was performed in this study to determine the antibacterial activity of gliding bacterial crude extract. Crude extracts were tested for antibacterial activity against seven pathogenic bacteria: *Vibrio cholerae* non O1/non O139 DMST 2873, *Listeria monocytogenes* DMST 1327, *Escherichia coli* DMST 4212, *Bacillus cereus* DMST 5040, *Salmonella typhimurium* DMST 562, *Staphylococcus aureus* DMST 8840 and *Serratia marcescens* TISTR 1354. Tested strains were activated twice in NB broth (at 37 °C, 18 h) and prepared to a turbidity of 0.5 McFarland standards (approximately 10^5^–10^6^ CFU/ml) as an inoculum. One milliliter of an inoculum was well poured into 9 ml of Muller-Hinton soft medium (0.75 % agar, w/v) in a Petri dish. While those dishes were being solidified, six wells (6 mm in diameter) were drilled into each plate. Methanolic crude extracts were prepared to a final concentration of 25 mg/ml, and aliquots (100 µl) were tested by filling each well. DMSO and tetracycline were used as the negative and positive controls, respectively. Tested plates were incubated at 37 °C for 24 h and then monitored for inhibitory clear zones around the wells. The test for each crude extract against each pathogenic strain was carried out three times. The antibacterial activity was determined as the minimal inhibitory concentration (MIC) value, which is the minimum concentration of the extract that could inhibit the growth of tested microorganisms.

### Nucleotide sequence accession numbers

The GenBank/EMBL/DDBJ accession numbers for the partial 16S rRNA gene sequences of the strains were RPD001, RPD008, RPD018, RPD027, and RPD049 are AB872448, AB872449, AB872450, AB872451, and AB872452, respectively.

## Results

### Isolation of aquatic gliding bacteria

Five isolates of aquatic gliding bacteria including strains RPD001, RPD008, RPD018, RPD027, and RPD049 were isolated from 54 specimens collected from Cheow Lan Reservoir. Although many types of specimens were used in the attempt to isolate gliding bacteria, only biofilms, plastics, and plant debris were found to provide the bacteria. Biofilms and plastics were the prolific sources of the aquatic gliding bacteria for this study. Two strains including RPD001 and RPD027 were isolated from biofilms. Also, RPD008 and RPD018 were obtained from plastics. In addition, one isolate (RPD049) was from plant debris. During the isolation program with four different isolation media, two strains of aquatic gliding bacteria (RPD027 and RPD049) were isolated using a DW medium, and two strains (RPD001 and RPD018) were obtained from a Vy/2 medium. One strain (RPD008) was achieved from a SAP2 medium (Table 1). However, no strains were isolated by modified MA medium.

**Table 1:** The isolation result of aquatic gliding bacteria

Strain	RPD001	RPD008	RPD018	RPD027	RPD049
Type of specimen	Biofilms	Plastic	Plastic	Biofilms	Plant debris
Isolation medium	Vy/2	SAP2	Vy/2	DW	DW

RPD is Cheow Lan Reservoir, where specimens were collected. Vy/2 is medium contained baker’s yeast paste. SAP2 is medium contained tryptone and yeast extracts. DW is a medium containing only distilled water

### Morphological, physiological and biochemical characteristics

**Fig. 1:**
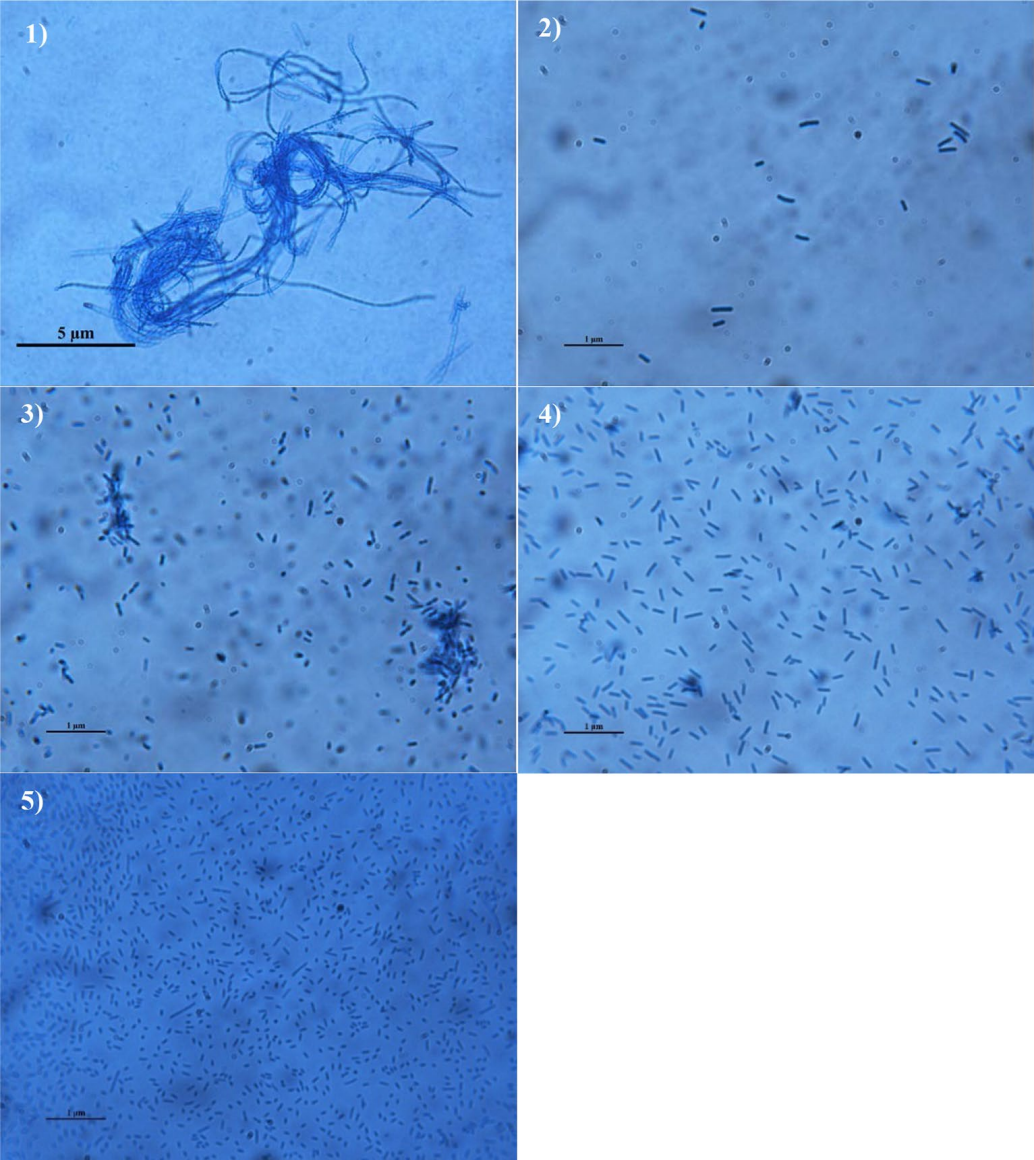
Cell morphology of aquatic gliding bacterial strains **1** RPD001 (*bar* 5 µm), **2** RPD008, **3** RPD018, **4** RPD027, and **5** RPD049 (2–5, *bars* 1 µm)

All five strains were Gram negative, aerobic, non-fruiting body, non-flagellate gliding bacteria. Strain RPD001 was filament shaped and 5–10 µm in length, while other four strains were shaped as short rods, ranging from 0.2 to 0.6 µm in length (Fig. 1). Swarm colony colors of strains RPD001, RPD008, and RPD018 were yellow, whereas strains RPD027 and RPD049 were pale-bright. The phenotypic, morphological, physiological and biochemical features of the isolates are summarized in Table 2. Some characterization of RPD008 was described here because its 16S rRNA sequence data showed a low similarity value to neighbor species (see below), indicating that it might be possible to propose as a novel genus. The main characteristics of RPD008 were described: its growth occurred under a 20–40 °C temperature range, with optimal growth observed at 30–35 °C. Tryptone and yeast extract were useful nitrogen sources for growth. Enzyme activities were positive for esterase (C4), esterase lipase (C8), and naphthol-AS-BI-phosphohydrolase. Acid production was produced from glycerol, D-arabinose, ribose, glucose, fructose, cellobiose, maltose, sucrose, starch, raffinose and L-fucose. Hydrolysis production was only positive for urea. RPD008 was isolated from plastics collected from Cheow Lan Reservoir, Surat Thani province, Southern Thailand.

**Table 2:** Phenotypic characteristics of the aquatic gliding bacterial isolates

Characteristic	RPD001	RPD008	RPD018	RPD027	RPD049
Cell morphology	Filament	Rod	Rod	Rod	Rod
Size in length (µm)	5–10	0.2–0.5	0.2–0.4	0.2–0.4	0.2–0.6
Colony color	Yellow	Yellow	Yellow	Pale-bright	Pale-bright
Gliding motility	+	+	+	+	+
Gram’s stain	Negative	Negative	Negative	Negative	Negative
Oxidase	–	–	–	–	–
Catalase	–	–	–	+	–
Enzyme activities					
Alkaline phosphatase	+	–	+	+	–
Esterase (C4)	+	+	+	+	+
Esterase lipase (C8)	+	+	+	+	+
Lipase (C14)	–	–	–	+	–
Acid phophatase	+	–	–	+	–
Naphthol-AS-BI-phosphohydrolase	+	+	+	+	+
β-Galactosidase	–	–	+	–	–
Hydrolysis of					
Arginine	+	–	–	+	–
Sodium citrate	+	–	–	+	–
Urea	–	+	+	+	+
Tryptophan	–	–	–	+	–
Sodium pyruvate	+	–	–	+	–
Kohn’s gelatin	+	–	–	–	–
Acid production of					
d-Arabinose	–	+	+	–	–
l-Arabinose	+	–	–	–	–
Ribose	+	+	+	–	+
d-Xylose	+	–	–	+	–
Galactose	+	–	–	–	–
Glucose	–	+	+	–	+
Fructose	+	+	+	–	+
Mannose	+	–	+	–	–
Mannitol	+	–	–	–	–
Esculin	–	–	–	+	+
Cellobiose	+	+	+	–	+
Maltose	+	+	+	–	+
Sucrose	+	+	+	–	+
Starch	+	+	+	–	+
Raffinose	+	+	+	–	–
d-Fucose	+	–	–	+	+
l-Fucose	–	+	+	–	–
5-Keto-gluconate	–	–	+	–	+
Glycerol	+	+	+	–	+

RPD is Cheow Lan Reservoir, where specimens were collected

## 16S rRNA gene sequence and phylogenetic analyses

**Fig. 2:**
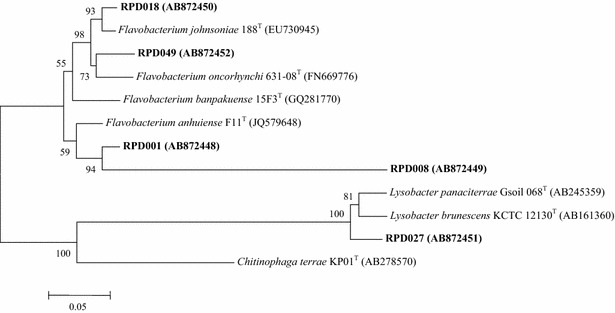
Phylogenetic tree of partial 16S rRNA gene sequences of aquatic gliding bacteria isolated from Cheow Lan Reservoir and their neighbor species (*bar* 0.05)

Phylogenetic analysis of the partial 16S rRNA gene sequence data of the aquatic gliding bacterial strains placed them all into one of two distinct phyla: *Bacteroidetes*, which belongs to family *Flavobacteriaceae* within the genus *Flavobacterium*; and *Proteobacteria*, which belongs to the family *Xanthomonadaceae*, within the genus *Lysobacter*. A blast search with the 16S rRNA gene sequences of strain RPD008 had similarity of 82 % to *Flavobacterium anhuiense* F11^T^ (JQ579648). Other strains RPD001 and RPD018, and RPD049 had similarity of 96 % to *F*. *anhuiense* F11^T^ (JQ579648) and *F*. *johnsoniae* 188^T^ (EU730945), respectively. Another strain RPD027 had similarity of 96 % to *Lysobacter brunescens* KCTC 12130^T^ (AB161360) (Fig. 2). On the basis of the16S rRNA gene sequencing, the strain RPD008 showed a noteworthy sequencing similarity value of less than 90 % and the phylogenetic tree formed a distinct lineage separating from *F*. *anhuiense* F11^T^ (Fig. 2).

### Antibacterial activity

**Fig. 3:**
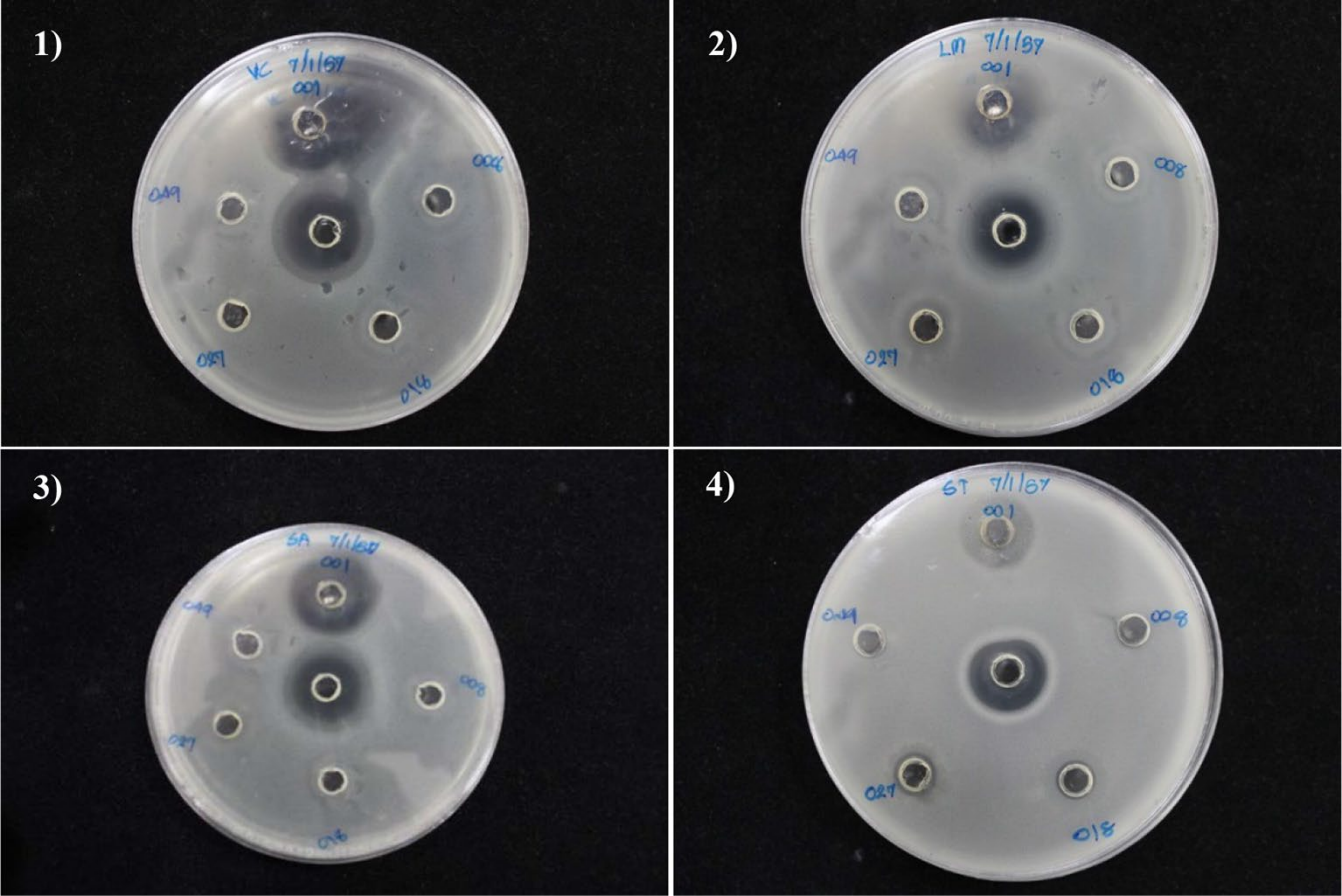
Inhibitory clear zone on agar-well diffusion plates of crude extract obtained from strain RPD001 (*top well* of each plate) against **1** *Vibrio cholerae* non O1/non O139 DMST 2873, **2** *Listeria monocytogenes* DMST 1327, **3** *Staphylococcus aureus* DMST 8840, and **4** *Salmonella typhimurium* DMST 562. Tetracycline (*central well* of each plate) was used as a positive control

Methanolic crude extracts of all five gliding bacterial strains were examined for antibacterial activity against pathogenic bacteria including three Gram positive bacteria (*B*. *cereus*, *L*. *monocytogenes*, and *S*. *aureus*) and four Gram negative bacteria (*E*. *coli*, *S*. *typhimurium*, *S*. *marcescens*, and *V*. *cholerae*). Antibacterial assay showed that crude extracts produced from four gliding bacteria strains (RPD008, RPD018, RPD027, and RPD049) exhibited no inhibitory clear zone against all tested pathogenic strains. It was noted that only another crude extract obtained from strain RPD001 (96 % similarity with *Flavobacterium anhuiense*) exhibited an inhibitory clear zone (Fig. 3). The obtained extract showed strong inhibitory activity against both Gram positive and Gram negative pathogenic bacteria including *L*. *monocytogenes* (MIC 150 µg/ml), *S*. *aureus* (MIC 75 µg/ml), and *V*. *cholerae* (MIC 300 µg/ml) (Table 3). However the extract exhibited a weak inhibitory effect on *Salmonella typhimurium* (MIC > 300 µg/ml) (Fig. 3).

**Table 3:** Antibacterial spectra of crude extract produced from RPD001 against pathogenic strains

Test pathogenic bacteria	MIC (µg/ml)
Gram positive	
*Bacillus cereus* DMST 5040	>300
*Listeria monocytogenes* DMST 1327	150
*Staphylococcus aureus* DMST 8840	75
Gram negative	
*Escherichia coli* DMST 4212	>300
*Salmonella typhimurium* DMST 562	>300
*Serratia marcescens* TISTR 1354	>300
*Vibrio cholerae* non O1/non O139 DMST 2873	300

MIC is the minimal inhibitory concentration

## Discussion

In this study, biofilms and plastics yielded the highest numbers of aquatic gliding bacteria. Biofilms are consortium layers of microorganisms occurring on the surfaces of most submerged substrates (Kadouri and O’Toole 2005). Hence, specimens collected from a submerged plastic surface covered with biofilms should be defined as ‘biofilms on plastic’. Many reports have indicated that biofilms are good sources for isolation of gliding bacteria (Donachie et al. 2004; Kwon et al. 2006; Rickard et al. 2005; Sangnoi et al. 2009; Vandecandelaere et al. 2009). The reasons for this may be because gliding bacteria play an important role in the biodegradation of organic compounds such as exopolysaccharides, which is commonly found in biofilms (Barbeyron et al. 2001; Johansen et al. 1999), and/or because gliding bacteria are ‘pioneer’ species that attach to substrates before development into fully-functioning biofilms. The isolation results of the gliding bacteria under study may depended on the composition of the isolation media, which is the nutrient source for gliding bacteria. DW medium is a growth-limiting nutrient containing only agar and distilled water. This medium might help to eliminate fast-growing species while enriching the relatively slow-growing gliding bacteria. However, it was time-consuming to obtain pure culture from this media, which is also subject to the risk of attack by fungi. In a previous study, our group used another growth-limiting nutrient medium known as SWG medium, which contains only sodium glutamate, sodium chloride, agar and distilled water, to isolate marine gliding bacteria. We found that a SWG medium was effective for marine gliding bacteria isolation (Hosoya et al. 2006, 2007, Sangnoi et al. 2008, 2009; Srisukchayakul et al. 2007). In this study we tried to design a new simple medium (DW) for isolation of fresh water gliding bacteria. We expected that a growth-limiting nutrient medium like DW would be suitable for isolation of slow-growing gliding bacteria that inhabit fresh water environments. In contrast, the Vy/2 medium contained a baker’s yeast paste (yeast cells), which released organic nutrients like amino acids, proteins, carbohydrates, fatty acids, vitamins, minerals, and other growth factors after autoclaving. This enrichment medium was suggested for fast-growing gliding bacteria isolation. However, these organic components are not only required by fast-growing gliding bacteria, but also by other bacterial contaminants. Thus, this medium enables both gliding bacteria and unwanted bacteria to flourish. As a result, an increased frequency of repeated subculturing is required to isolate the gliding bacteria strains under study from the contaminants. The 16S rRNA gene sequence analysis of RPD008 showed less than 90 % similarity value and the phylogenetic tree exhibited clearly separated lineage from related species. These results indicate that the strain RPD008 should be characterized as a novel genus, differentiated from *Flavobacterium*. However, determination of more chemotaxonomic characteristics such as cellular fatty acid profile, respiratory quinone and G+C content should be the subject of further study. Likewise, three strains (RPD001, RPD018 and RPD049) of genus *Flavobacterium* and one strain (RPD027) of genus *Lysobacter* may have the opportunity to be proposed as new species with 96 % similarity value. However, DNA–DNA hybridization experiments with closely-related species of candidate strains must be performed to confirm this presumption. As for antibacterial activity, only crude extract obtained from strain RPD001 was found to show inhibitory effects against pathogenic bacteria. Bioactive compounds composed in this crude extract may have had broad activity because they inhibited both Gram positive and Gram negative pathogenic bacteria. The result suggested the possibility that crude extract produced from strain RPD001 could be developed for use as a broad antibiotic agent. However, the structure elucidation of bioactive compounds and other biological assay of this strain also require further study. In this study, crude extracts obtained from other strains showed no significant antibacterial activity. However, several antibiotics isolated from *Flavobacterium* and *Lysobacter* have been reported by other researchers (Bonner et al. 1988; Hashizume et al. 2001; Islam 2010; Li et al. 2008; O’Sullivan et al. 1988; Park et al. 2008; Singh et al. 1982; Yagi and Maruyama 1999). We suggest that these crude extracts may exhibit other biological activities if more biological assays are conducted.

## Conclusion

The study results indicated that biofilms on substrates were the good source for aquatic gliding bacteria isolation. Growth-limiting nutrient medium (DW) was suitable for slow-growing gliding bacteria, while a more nutrient-rich medium (Vy/2) was suitable for fast-growing gliding bacteria. Strain RPD008 should be classified as a novel genus due to its low similarity value. Crude extract produced from strain RPD001 showed inhibitory effects against Gram positive and Gram negative pathogenic bacteria; it could possibly be developed as a broad antibiotic agent.
